# Local Voronoi Computation and Sweep Line Intersection for Efficient Voronoi-Based Grouping Test Localization in Wireless Sensor Networks

**DOI:** 10.3390/s26206397

**Published:** 2026-10-10

**Authors:** Goran Đambić, Patrik Perčinić, Ivana Ogrizek Biškupić, Aleksander Radovan

**Affiliations:** Algebra Bernays University, Gradišćanska 24, 10000 Zagreb, Croatia; ppercin@algebra.hr (P.P.); ivana.ogrizekbiskupic@algebra.hr (I.O.B.); aleksander.radovan@algebra.hr (A.R.)

**Keywords:** wireless sensor networks, node localization, range-free localization, Voronoi diagram, sweep line algorithm, computational efficiency, simulation

## Abstract

**Highlights:**

**What are the main findings?**

- Computing each Voronoi diagram from only the *k* nearest anchor nodes (instead of all anchors in a group) reduces the Voronoi-phase time of the VTL localization algorithm by 69–83%, with an accuracy penalty below 2% for *k* ≥ 10 on average across network sizes.
- Replacing the grid scan with an exact sweep line algorithm accelerates the positive-region intersection phase by 7.7–18.1% while producing identical localization results; together, the two optimizations cut total execution time by 37–61%.

**What are the implications of the main findings?**

- The optimized VTL algorithm scales to large and dense sensor networks and to resource-constrained hardware without sacrificing localization quality.
- The neighborhood size must be chosen deliberately: *k* = 10–12 is the recommended operating range, whereas *k* = 6 degrades both accuracy and overall speed.

**Abstract:**

Node localization is a fundamental service in wireless sensor networks (WSNs), and range-free schemes such as the Voronoi diagram-based grouping test localization (VTL) achieve good accuracy without distance measurements. However, VTL repeatedly constructs Voronoi diagrams over all anchor nodes of each group and resolves the intersection of positive regions by an exhaustive grid scan, which limits its scalability. This paper proposes and evaluates two optimizations: (1) a local Voronoi computation that restricts the computation to the *k* anchors nearest to the strongest-signal anchor (including that anchor itself), and (2) an exact sweep line algorithm that replaces the grid scan in the intersection phase. Both were evaluated in a purpose-built, reproducible simulator through 5184 primary and 464 supplementary simulations spanning 60–1000 nodes, two deployment areas (200 m and 400 m squares) and *k* = 6–20, with paired Wilcoxon signed-rank tests. The local computation reduced the Voronoi-phase time by 69–83%, with the accuracy penalty falling below 2% for *k* ≥ 10 on average across network sizes, while the percentage of localized nodes remained unchanged. The sweep line algorithm reduced the intersection-phase time by 7.7–18.1% while producing identical localization results. Combined, the two optimizations reduced the total execution time by 37–61% (up to 4.7-fold at 1000 nodes). The conclusions are robust across anchor densities of 20–70%, ten independent seeds, and log-normal shadowing noise in the anchor selection. We recommend *k* = 10–12 as the default operating range, whereas *k* = 6 degrades both accuracy and overall speed.

## 1. Introduction

Wireless sensor networks (WSNs) are one of the fundamental technologies for collecting data from the physical world in real time. Much of their usefulness depends on the ability to accurately determine the position of each sensor node, a process known as localization: without positional information, the collected data lack context and lose much of their value. Although equipping every node with a global positioning system (GPS) receiver is an obvious solution, this approach is economically and technically impractical in large, widely distributed networks due to the cost of GPS modules, their energy consumption, susceptibility to jamming, and unreliable reception indoors or underground [1].

A WSN consists of nodes that traditionally only collected sensor readings and forwarded them onwards. Today’s energy-efficient, fast and inexpensive microprocessors allow increasingly complex functionality to be implemented directly on the nodes, which are therefore no longer limited to data collection and forwarding but can also perform local analysis, recognize patterns and make decisions in real time [2,3].

Anchor nodes are nodes whose exact locations are known and which serve as reference points in the system. Their location can either be recorded at deployment time or obtained from an on-board GPS module. GPS-equipped nodes, although precise, increase the initial cost, add potential points of failure, can be jammed, and consume more energy. In practice, the number of GPS-equipped nodes is therefore kept to a minimum and combined with localization methods that determine locations indirectly [4]. Unknown nodes are nodes whose locations are not known in advance; they are not equipped with GPS modules and cannot determine their positions independently. To do so, they must be able to communicate with anchor nodes in their vicinity: by collecting positional information from the anchors and applying a localization algorithm, they can estimate their approximate location with varying precision [4].

Localization algorithms in WSNs can be divided into two groups: range-based algorithms, which rely on measured distances or angles, and range-free algorithms, which do not require any distance measurements [5,6,7].

In range-based algorithms, the position of an unknown node is determined using various measurement techniques. Received signal strength indication (RSSI) estimates the distance to an anchor node from the strength of its received signal and a model of signal propagation [8,9]. Angle of arrival (AoA) measures the angles at which signals arrive from anchor nodes and estimates the position by triangulation [10]. Time difference of arrival (TDoA) computes distances from the difference in arrival times of two signals with different propagation speeds (e.g., radio and ultrasound) emitted by the same anchor [11], while time of arrival (ToA) directly measures the arrival time of a signal of known speed, the technique used by GPS [12].

Range-free algorithms instead exploit topological information about the network. In DV-Hop (distance vector hop), anchor nodes flood the network with their coordinates accompanied by a hop counter that each forwarding node increments; every node thus learns the minimum hop count to each reachable anchor and approximates its own location from these counts and an estimated distance per hop [13,14]. The Hop-Terrain approach is similar: the unknown node first estimates its position from the average distance per hop and then iteratively refines the estimate using a least-squares method [15]. In the Centroid System, an unknown node places itself at the centroid of the coordinates of all anchor nodes whose signals it receives, which makes the method strongly dependent on anchor density and distribution [16,17]. In APIT (approximate point in triangulation), the deployment area is divided into overlapping triangles defined by anchor positions; the unknown node performs point-in-triangulation tests with different combinations of anchors, and the final estimate is the centre of gravity of the area in which the node most probably resides [18]. In the Gradient algorithm, nodes propagate anchor positions together with hop counts, and the unknown node estimates its distances to multiple anchors and determines its position by multilateration, minimizing the difference between the estimated and actual distances [19].

Research on range-free localization remains active. Numerous recent studies improve the DV-Hop family through better anchor selection or metaheuristic optimization (including optimum anchor-node subsets [20], adaptive genetic algorithms [21], swarm-intelligence approaches [22] and RSSI-based hop-distance corrections [23]), or explicitly target the energy and message cost of localization on resource-constrained nodes [24,25], while machine-learning models trained on connectivity data have also been proposed [26,27]. In parallel, Voronoi diagrams and related computational-geometry structures are an established tool in WSNs for coverage and deployment problems [28,29,30,31], including local Voronoi constructions for sensor deployment in complex environments [29] and Voronoi-based coverage and energy analysis of mobile sensors in obstructed indoor environments [31], and Voronoi partitioning has recently been applied to three-dimensional localization [32]. Most of these efforts, however, focus on improving localization accuracy or communication cost, whereas the computational cost of constructing and querying the geometric structures that underlie Voronoi-based localization has received comparatively little attention. This paper addresses precisely that gap in the context of the VTL scheme.

A prominent representative of the range-free group is the Voronoi diagram-based grouping test localization (VTL) scheme [33,34]. Its underlying idea is to use Voronoi diagrams to determine the region in which an unknown node may reside, and then to progressively narrow this region down by combining the results obtained from several groups of anchor nodes. All anchor nodes periodically broadcast messages containing the exact locations of all anchors in the network, while the unknown node passively listens and collects these messages. Upon receiving a message, the unknown node marks its sender as detectable, i.e., the unknown node is within that anchor’s communication range. Since every message carries the complete anchor map, receiving even a single message provides the unknown node with the locations of all anchor nodes in the network. At the end of this phase, the unknown node therefore holds a list of all anchors divided into two sets: those it can detect and those that are too far away [33].

In the second phase, the unknown node iteratively forms *K* groups, each containing approximately one half of the anchor nodes. In every iteration, two detectable anchors are selected at random to seed two new groups, and all remaining anchor nodes (both detectable and undetectable) are then randomly assigned to one of them. The procedure is repeated until *K* groups have been formed, which implies that *K* must be an even number no smaller than two. This phase, like all others, runs independently in every unknown node, so the resulting groups are specific to each node [33].

**Figure 1:**
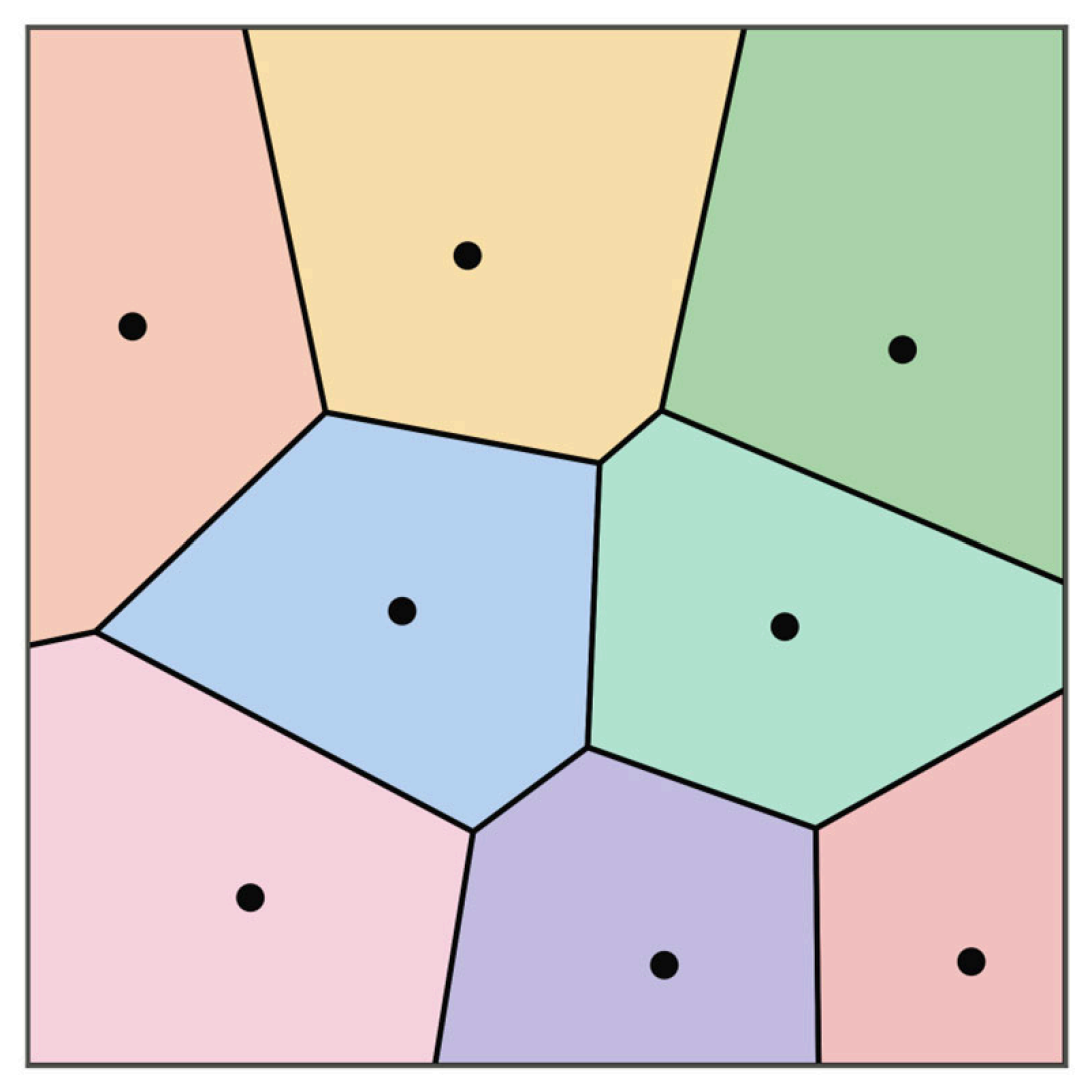
Example of a Voronoi diagram. Each cell contains all points of the plane that are closer to its node than to any other node. The cell colors are arbitrary and serve only to visually distinguish neighboring cells.

A Voronoi diagram partitions the plane into cells according to the distance from a predefined set of nodes. Each node is assigned one Voronoi cell, defined as the set of all points of the plane that are closer to that node than to any other node; the collection of all cells forms the Voronoi diagram [33,35]. Figure 1 shows an example of a Voronoi diagram of eight nodes.

**Figure 2:**
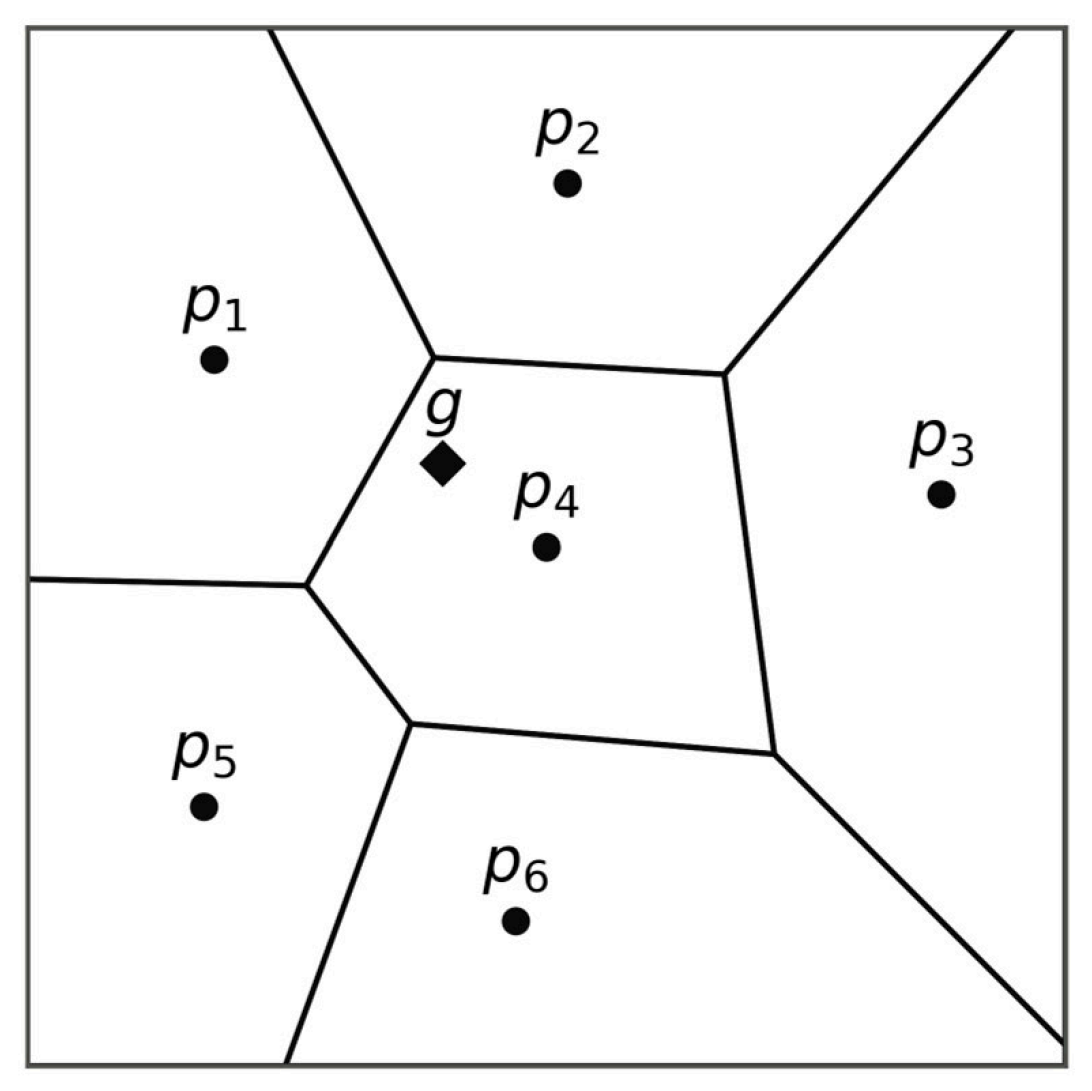
Voronoi diagram of one group of anchor nodes (*p*_1_–*p*_6_). The unknown node *g* lies in the cell of its nearest anchor *p*_4_, which is marked as the positive cell.

In the next phase of the VTL algorithm, the unknown node processes each of the *K* groups separately to determine the region in which it may reside according to that group alone. A Voronoi diagram is constructed over all anchor nodes of the group, dividing the space into cells in which each point is closer to its anchor than to any other anchor in the group. The unknown node then compares the RSSI values of all detectable anchors within the group and identifies the one with the strongest signal; the Voronoi cell belonging to that anchor is marked as the positive cell, and the algorithm assumes that the unknown node lies inside it. Importantly, the algorithm does not suffer from the problems characteristic of signal propagation models, because it never attempts to estimate a distance from the RSSI—it only determines which signal is the strongest. Figure 2 shows the Voronoi diagram of one group: the point *g* denotes the unknown node, while *p*_1_ to *p*_6_ denote the anchor nodes of the group. Since *g* is closest to *p*_4_, the cell of *p*_4_ would be marked as the positive cell in this example [33].

Repeating this procedure for all *K* groups yields *K* positive cells for each unknown node [33]. In the final phase, these *K* positive cells are used to determine a more precise location estimate. The positive cells will not always overlap perfectly, owing to occasional errors in selecting the nearest cell caused by signal interference and GPS positioning inaccuracies. For this reason, the original scheme does not use a simple geometric intersection but a grid scan method that acts as a voting system. The region of interest (the area around the unknown node encompassing all positive cells) is divided into a rectangular grid of cells of a predefined size, and each grid cell is assigned an integer weight initialized to zero. The algorithm iterates over the *K* positive regions and, for every grid cell, checks whether its center lies inside the current positive region: if it does, the weight is incremented by one; otherwise, it is decremented by one. After all positive regions have been processed, the maximum weight is identified, and the set of all grid cells attaining this maximum constitutes the estimated region. The final location estimate of the unknown node is computed as the centroid of the centers of these cells, which completes the localization procedure [33].

An analysis of the original scheme [33] reveals two computationally expensive steps. The first is the repeated construction of a Voronoi diagram for every group of anchor nodes. The original algorithm uses a global approach: all anchor nodes of a group enter the computation, regardless of their distance from the unknown node. However, the shape and boundaries of the Voronoi cell nearest to the unknown node depend exclusively on its immediate neighbors. Including hundreds or thousands of distant anchors from the same group does not contribute to the accuracy of the relevant cell’s shape, but it does increase the computational load. In dense networks, where each group may contain a large number of nodes, this step becomes a potentially unnecessary drain on computational resources.

**Figure 3:**
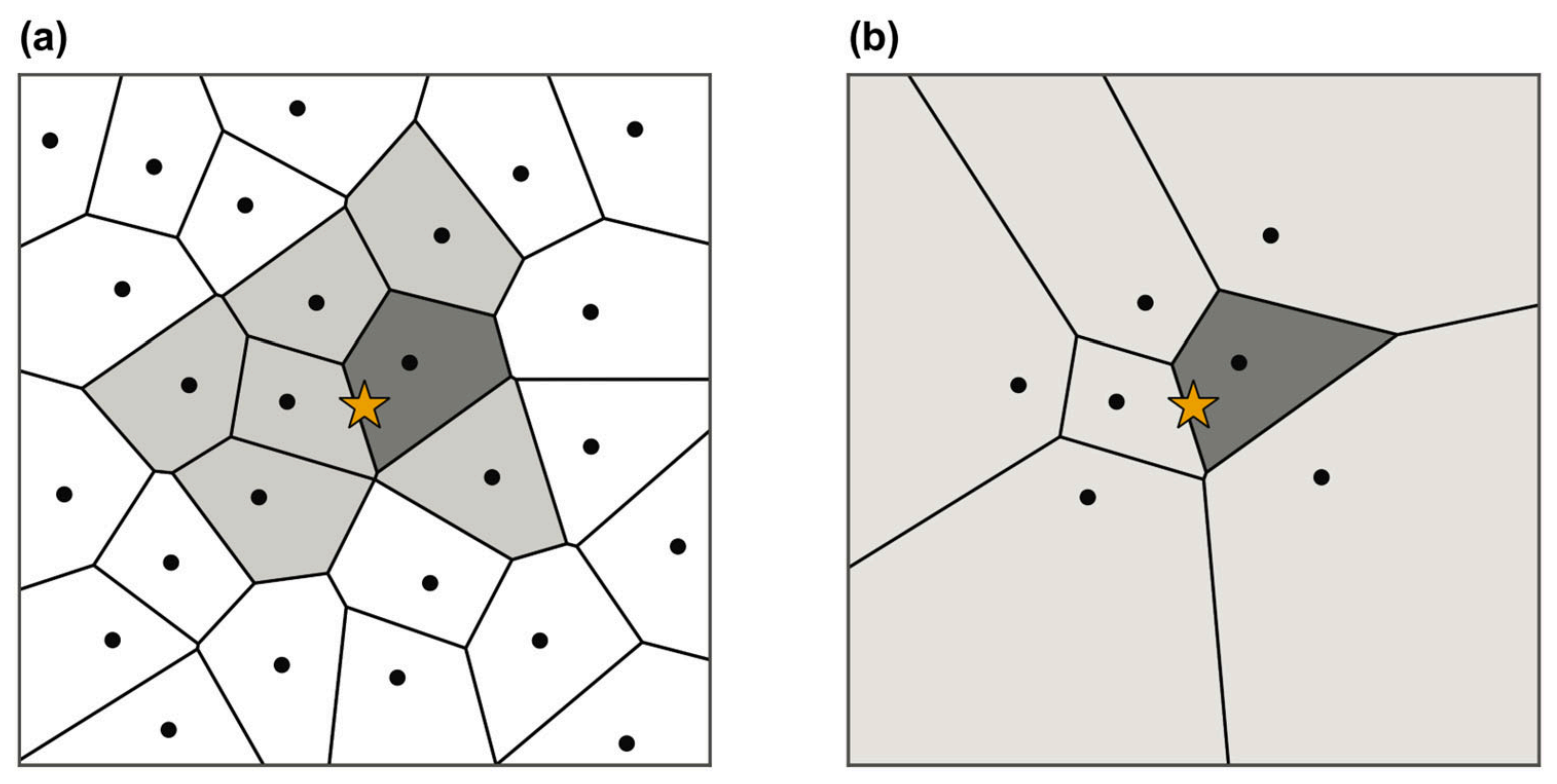
Motivation for the local Voronoi computation: (**a**) global Voronoi diagram of all anchor nodes of a group: the star marks the unknown node, the dark grey cell belongs to its nearest anchor, and the light grey cells are its direct neighbors; (**b**) Voronoi diagram constructed from only the nearest anchor and its closest neighbors: the shape of the nearest cell remains unchanged.

Figure 3 illustrates this observation. Figure 3a shows the full Voronoi diagram of all anchor nodes of a group: the star marks the location of the unknown node, the dark grey cell belongs to its nearest anchor, and the light grey cells share an edge with that cell. Figure 3b shows the diagram constructed from only the nearest anchor and its closest neighbors: the shape of the relevant (dark grey) cell is unchanged, because anchors whose cells do not border it have no influence on its geometry.

The second computationally expensive operation is the grid scan algorithm, which divides the observed area into a rectangular grid and tests every cell of the grid for membership in the positive regions. Although conceptually simple, its efficiency depends directly on the chosen grid resolution: achieving high precision requires small grid cells, and the number of cells that must be tested grows quadratically as the cell size decreases. Likewise, enlarging the observed area increases the number of cells regardless of the number or density of nodes. This brute-force scan of the area is computationally demanding, and replacing it with a sweep line algorithm can be expected to yield better performance.

Sweep line algorithms are a family of computational geometry algorithms that move an imaginary line across the plane and, at any moment, process only the objects that the line currently touches, instead of considering the entire problem at once [36]. This substantially reduces complexity, because the algorithm never has to track all elements simultaneously. The approach was originally developed for the efficient solution of the line segment intersection problem [37] and was later applied to the construction of Voronoi diagrams in Fortune’s algorithm [38], which maintains a sweep line together with a “beach line” of parabolic arcs that jointly describe the current state of the diagram under construction.

These two computationally expensive operations hinder the scaling of the VTL algorithm to large networks and resource-constrained hardware, which leads to a clear research problem: how can the efficiency of the VTL algorithm be increased without compromising its localization accuracy? To examine a possible solution, this paper proposes two optimizations:

- A local Voronoi approach, which, instead of all anchor nodes of a group, restricts the Voronoi computation to the *k* anchors nearest to the strongest-RSSI anchor;
- A sweep line algorithm as a replacement for grid scanning, which processes only the cells intersected by the moving line instead of the entire grid.

For the purposes of this analysis, a simulator was developed that reproduces the original VTL algorithm and enables the evaluation of the proposed optimizations.

The aim of this work is to analyze the limitations of the existing VTL algorithm, implement the two proposed optimizations, and quantitatively compare the efficiency and accuracy of the optimized versions with the original algorithm. Based on previous research and a preliminary analysis, the following hypotheses are formulated:

**H1.**  *The local Voronoi approach significantly reduces the execution time of the VTL algorithm compared with the global approach.*

**H2.**  *Increasing the number of nearest neighbors (k) reduces the localization error but increases the execution time.*

**H3.**  *The sweep line algorithm significantly reduces the duration of the positive-region intersection phase compared with grid scanning, without increasing the localization error.*

**H4.**  *Both optimizations applied together enable a more scalable execution of the VTL algorithm while preserving its accuracy.*

In summary, the novelty of this work does not lie in the individual geometric techniques (nearest-neighbour-restricted Voronoi construction and sweep-based enumeration are classical tools of computational geometry [36,37,38]) but in their integration into the VTL scheme with explicit correctness guarantees, and in the resulting evidence base. To the best of our knowledge, this is the first work that treats the computational cost of the geometric machinery of Voronoi-based localization (rather than localization accuracy, energy or message cost) as the primary object of study. Specifically, the paper contributes: (1) a local Voronoi computation with a quantified geometric rationale linking the neighbourhood size k to the Delaunay neighbours of the strongest-signal anchor; (2) a sweep-line replacement of the grid scan that is provably exact, producing identical localization results; (3) a statistically rigorous, fully reproducible evaluation (5184 paired primary simulations with open code and data, complemented by noise, anchor-density and seed-stability campaigns) establishing total-time reductions of 37–61% with an accuracy penalty below 2% at the recommended settings; and (4) actionable, density-aware guidance for choosing k, including the finding that an overly aggressive reduction (k = 6) degrades speed as well as accuracy.

The remainder of this paper is organized as follows. Section 2 describes the simulator, the experimental design and the statistical methodology. Section 3 presents the results: the validation of the simulator against the behavior of the original scheme, the evaluation of each optimization separately, their combined effect, and the analysis of the choice of *k*. Section 4 discusses the findings in the context of the four hypotheses, together with practical implications and limitations, and Section 5 concludes the paper.

## 2. Materials and Methods

This work experimentally evaluates the efficiency of two optimizations proposed for the VTL algorithm [33]: (1) the local computation of the Voronoi diagram and (2) the sweep line algorithm for determining the intersection of positive regions. This section describes the simulator, the experimental design, the measured variables and the statistical analysis.

### 2.1. The Proposed Optimizations

For every group of anchor nodes, the original scheme constructs a Voronoi diagram over all *m* anchors of the group, although only the cell of the strongest-signal anchor is subsequently used. The local computation (Algorithm 1) replaces this global construction as follows. Let a* denote the detectable anchor of the group with the strongest received signal. The unknown node computes the distances from a* to all anchors of the group and constructs the Voronoi diagram over the *k* anchors nearest to a* (a set that includes a* itself); if the group contains at most *k* anchors, the global diagram is used. The positive cell is then the cell of a* in this local diagram, clipped to the deployment area exactly as in the original algorithm. The rationale is geometric: the shape of the cell of a* is determined exclusively by its Delaunay neighbors, so the local cell is identical to the global one whenever all Delaunay neighbors of a* are among the selected anchors, and the approximation error vanishes as *k* grows. This geometric argument can be made quantitative. By Euler’s formula for planar triangulations, the average number of Delaunay neighbours of a site is below six regardless of the number of anchors in the group [35]. In a Monte Carlo analysis of the deployment geometries used in our experiments (uniformly random anchors, group sizes *m* = 40–200 corresponding to *N* = 200–1000, 2000 random configurations each), the strongest-signal anchor a* had on average 5.5–6.1 Delaunay neighbours (95th percentile: 8, maximum: 12). The *k* anchors nearest to a* captured on average 65–72% of these neighbours for *k* = 6, 86–90% for *k* = 10, and 91–94% for *k* = 12, and the local cell of a* coincided exactly with the global one in 8–14% of configurations for *k* = 6, 44–58% for *k* = 10, and 61–71% for *k* = 12. This quantifies the theoretical basis of the neighbourhood size: *k* = 6 leaves at most five candidate neighbours and therefore cannot even in principle cover the typical six Delaunay neighbours, which explains its pathological behaviour observed in Section 3, whereas for *k* = 10–12 the anchors missed are predominantly distant neighbours whose omission clips only marginal parts of the cell; the residual error is further attenuated by the aggregation of the *K* positive regions in the intersection phase. Because the expected number of Delaunay neighbours does not grow with anchor density, a fixed *k* remains applicable across densities, although the slightly lower coverage at larger group sizes is consistent with the density dependence of the accuracy penalty reported in Section 3.6. The analysis script is available together with the rest of the code (see the Data Availability Statement).
**Algorithm 1.** Local Voronoi computation for one group of anchor nodes.Input: anchor positions P = {p1, …, pm} of the group; strongest-signal        anchor a* ∈ P; neighborhood size k; deployment area B Output: positive cell C(a*) 1 if m ≤ k then 2      V ← VoronoiDiagram(P)   ▷ fallback: global diagram 3 else 4      for i ← 1 to m do d_i ← ||p_i − a*||^2^ 5      P_k ← the k points of P with the smallest d_i   ▷ includes a* (d = 0) 6      V ← VoronoiDiagram(P_k) 7 C(a*) ← cell of a* in V, clipped to B 8 return C(a*)

In terms of asymptotic cost per group, the neighbor selection requires computing *m* distances and sorting them, *O*(*m* log *m*) in our implementation (a linear-time selection or a spatial index would reduce this further), while the cost of the diagram construction itself drops from *O*(*m* log *m*) to *O*(*k* log *k*) [36]. In practice the dominant saving lies elsewhere: the per-cell post-processing (reconstructing finite cell polygons and clipping them to the deployment area) is linear in the number of cells and therefore drops from Θ(*m*) to Θ(*k*) polygons per group. Since the procedure is repeated for each of the *K* groups of every unknown node, the saving multiplies across the network; Section 3.2 quantifies it empirically.

The second optimization targets the intersection of the *K* positive regions. In our simulator, both intersection algorithms operate on the same regular grid of cell size l, restricted to a square window of side 4*R* centered on the unknown node (where *R* denotes the fixed communication radius). Every positive region contains the unknown node by construction (the node is closer to the selected anchor than to any other anchor of the group), so the window captures the region intersection around the node; the same window is used by both algorithms, and any truncation of distant parts of a region therefore affects them identically. Grid scanning clips each positive region to the window and then tests every grid cell of the region’s bounding box for intersection with the region: the number of tests is proportional to the area of the bounding box, regardless of the region’s actual shape. The sweep line variant (Algorithm 2) instead advances a vertical strip of width l across the bounding box, intersects the region with the strip, and tests only the rows spanned by the resulting slice. Empty parts of the bounding box are never touched, and each test is performed against a smaller clipped polygon.
**Algorithm 2.** Column-strip sweep for the positive-region intersection.Input: positive regions A_1, …, A_K; grid cell size l; window W of side 4R        centered on the unknown node; weight grid w initialized to zero Output: location estimate 1 for j ← 1 to K do 2     Q ← A_j ∩ W; if Q = ∅ then continue 3     for each grid column c intersecting the bounding box of Q do 4        S ← Q ∩ strip(c)   ▷ vertical strip of width l at column c 5        for each connected piece S_p of S do 6            for each grid row r spanned by the y-extent of S_p do 7               if S_p intersects cell(c, r) then w[r][c] ← w[r][c] + 1 8 M ← set of grid cells attaining the maximum weight in w 9 return centroid of the centres of the cells in M

Crucially, the sweep line is an exact replacement, not an approximation. Both algorithms increment the weight of a grid cell if and only if the positive region intersects that cell: every cell of column *c* is contained in the strip of column *c*, so the slice *Q* ∩ strip(*c*) intersects a cell exactly when *Q* itself does. The two algorithms therefore produce identical weight matrices, hence identical maximum-weight regions and identical centroid estimates, for every unknown node; they differ only in the order in which cells are enumerated and in the number of cells that are needlessly tested. This is why the localization results in Section 3.3 agree pair by pair, and why the benefit of the sweep grows with the deployment area, where bounding boxes contain proportionally more empty cells.

The costs of the two enumeration strategies can also be stated formally. Let *b_c_* and *b_r_* denote the number of grid columns and rows of a positive region’s bounding box, and let *n_j_* be the number of grid cells actually intersected by region *A_j_*. Grid scanning evaluates the intersection predicate for every cell of the bounding box, i.e., *Θ*(*b_c_* · *b_r_*) predicate tests per region, irrespective of the region’s shape. The column-strip sweep performs *b_c_* polygon–strip intersections and then evaluates the predicate only for the cells within the y-extent of each slice, i.e., *O*(*b_c_* + $\sum_{c}r_{c}$) tests, where *r_c_* ≤ *b_r_* is the number of rows spanned by the slice in column c. Because the positive regions are convex (each is a Voronoi cell clipped to the deployment area and the window, an intersection of convex sets), every slice is a single connected piece whose y-extent tightly bounds the cells it can intersect, so $\sum_{c}r_{c}$ approaches *n_j_* and the sweep is output-sensitive: its cost is proportional to the number of cells the region actually covers, plus a term linear in the number of columns, whereas grid scanning always pays for the full bounding box. The two coincide in the worst case of a region that fills its bounding box, which explains why the advantage of the sweep grows with the deployment area (Section 3.3), where bounding boxes of elongated or slanted regions are mostly empty, and why it shrinks in small, densely covered areas (Section 3.6).

Three implementation details deserve note. First, the simulator accumulates a hit count *h* (the number of positive regions intersecting a cell) instead of the ±1 votes of the original scheme; since the number of groups *K* is fixed, the ±1 score equals 2*h* − *K*, a strictly increasing transform of *h*, so the maximum-weight region is identical. Second, both intersection algorithms use a region–cell intersection test rather than the cell-center test described in [33]; as the two compared algorithms share the same predicate, this choice does not affect any of the comparisons reported below. Third, the simulator forms each pair of groups by splitting the detectable and the undetectable anchors separately into random halves; a stratified variant of the random grouping of [33] that keeps the groups balanced and guarantees that at least one group of each pair contains a detectable anchor; the grouping procedure is shared by all evaluated configurations. These deviations do not compromise the validity of the reported comparisons, and their influence on accuracy and runtime is bounded as follows. The hit-count accumulation provably yields the same estimate as the ±1 voting of [33] for every node, since the two scores are related by the strictly increasing transform 2*h* − *K*. The region–cell intersection test is shared by both intersection algorithms, so it cannot influence any paired comparison; relative to the cell-center test described in [33] it makes a cell’s weight sensitive to partial overlaps, which may shift absolute weights (and thereby contribute, together with the idealized radio model, to the small absolute differences from [33] discussed in Section 3.1), but it does so identically for the baseline and the optimized variants, and its per-test cost is likewise marginally higher for both algorithms alike. The stratified grouping preserves the expected group composition of the original random halving while guaranteeing that every pair of groups can produce positive regions; it is executed once per node independently of the evaluated configuration, and its runtime share is negligible (below 1.6% of total execution time on average in all configurations).

### 2.2. Simulator, Experimental Design and Statistical Analysis

All experiments were performed with a purpose-built simulator that generates sensor networks, reproduces the original VTL algorithm and integrates the proposed optimizations. The simulator provides full control over the node distribution and the parameters of the localization algorithm, and guarantees reproducible execution through a fixed pseudo-random seed. It was developed in Python (v3.13.2) using the PyQt6 (v6.6 or later) and pyqtgraph (v0.13 or later) libraries for the graphical user interface and visualization, SciPy (v1.18.1) [39] and the foronoi library (v1.0.3) [40] for the construction of Voronoi diagrams, and Shapely (v2.1.2) [41] for geometric operations. Through its graphical interface, the simulator allows the parameters to be adjusted, the results to be visualized, the execution times of individual algorithm phases to be measured, batch runs to be launched, and the results to be exported in CSV format. Its source code is publicly available (see the Data Availability Statement). The experiments were designed to:

- reproduce the qualitative behavior of the original VTL algorithm;
- compare the optimized variants against the original algorithm;
- quantify the effects of the proposed optimizations on localization accuracy and execution time.

Four algorithm configurations were evaluated, corresponding to all combinations of two independent variables: the Voronoi computation mode (global, using all anchor nodes of a group, or local, using only the *k* anchors nearest to the strongest-RSSI anchor) and the positive-region intersection algorithm (grid scanning or sweep line).

For the local mode, the neighborhood size took the values *k* = 6, 8, 10, 12, 14, 16, 18 and 20. Networks of 60 to 1000 nodes were simulated in steps of 20 nodes (48 network sizes), with nodes deployed uniformly at random in a square area of either 200 m × 200 m or 400 m × 400 m. Each configuration was repeated three times, each with a different node deployment. The remaining parameters were fixed in all simulations: 40% of the nodes were anchor nodes, the communication radius was *R* = 20 m, the number of groups was *K* = 6, and the grid cell size was 2 m. All runs used the pseudo-random seed 4275, so that, for a given network size and repetition, every configuration operated on an identical node deployment; this makes all comparisons between configurations paired by design and every run exactly reproducible. In total, 5184 simulation runs were performed (2 areas × 48 network sizes × 3 repetitions × 18 configurations per area: 2 global and 2 × 8 local).

For every simulation run, the following dependent variables were recorded: the average normalized localization error *E_a_*, the percentage of localized nodes *P_e_*, and the execution times of the individual phases of the algorithm: the construction of the Voronoi diagrams, the computation of the positive-region intersection, and the total execution time. The average normalized localization error is computed as

(1) $$E_{a}=\frac{1}{N_{d}\cdot R}\sum_{i=1}^{N_{d}}\sqrt{{\left(x_{i}-{\hat{x}}_{i}\right)}^{2}+{\left(y_{i}-{\hat{y}}_{i}\right)}^{2}}$$

where *N_d_* is the number of unknown nodes that can be detected and localized, (*x_i_*, *y_i_*) are the true coordinates of node *i*, (${\hat{x}}_{i}$, ${\hat{y}}_{i}$) are its estimated coordinates, and *R* is the communication radius. Normalizing by *R* makes the error dimensionless and comparable across deployment areas. The percentage of localized nodes is computed as

(2) $$P_{e}=\frac{N_{d}}{N_{u}}\times 100$$

where *N_u_* is the total number of unknown nodes in the network. Three comparisons were derived from the four configurations: Experiment 1 evaluates the local against the global Voronoi computation (with grid scanning fixed), Experiment 2 evaluates the sweep line algorithm against grid scanning (with the global Voronoi computation fixed), and Experiment 3 evaluates both optimizations combined against the original algorithm.

All simulations were executed on a single workstation equipped with an Intel Core Ultra 9 185H CPU (Intel, Santa Clara, CA, USA; 16 cores, up to 5.1 GHz) and 64 GB of RAM, running Microsoft Windows 11 Enterprise (Microsoft, Redmond, WA, USA), under CPython 3.13.2 with NumPy 2.5.2, SciPy 1.18.1, Shapely 2.1.2 and foronoi 1.0.3; the simulator executes in a single thread, and all phase durations were measured with Python’s high-resolution time.perf_counter timer. The reported Voronoi-phase time covers the complete phase as executed by each variant: for the local variant it includes the computation of the distances from the strongest-signal anchor to all anchors of the group, the sorting-based selection of its *k* nearest neighbours, the construction of the Voronoi diagram over the selected anchors, and the post-processing shared by both variants (clipping of infinite cells and identification of the positive region). The overhead of the neighbour search and sorting is therefore fully contained in the reported speed-ups. The relative comparisons were additionally sanity-checked with a deterministic profiler (cProfile): in both the baseline and the fully optimized configuration approximately 60% of the execution time is spent in the same compiled geometric primitives of the Shapely library, our Python code accounts for about 15% in both, and the speed-up manifests as a reduction in the number of calls to identical primitives (for example, 4.4× fewer region–cell intersection calls at *N* = 600) rather than as a shift in where time is spent. The reported speed-ups therefore reflect genuine reductions in operation counts rather than asymmetric Python overhead.

Descriptive statistics (means, standard deviations and 95% confidence intervals) were computed for all metrics, separately for every combination of configuration, network size and deployment area. Relative improvements are reported as the mean, over all network sizes, of the per-size ratios between the optimized and the baseline configuration, averaged over the three repetitions.

Because all configurations operate on identical node deployments for a given network size and repetition, all comparisons between configurations are paired. Differences were therefore tested with two-sided paired Wilcoxon signed-rank tests over the *n* = 144 (network size, repetition) pairs per deployment area. The non-parametric test was chosen because Shapiro–Wilk tests showed that the paired differences deviate substantially from normality, as execution times scale strongly with network size. Effect sizes are reported as Cohen’s *d_z_* computed on the paired differences, with negative values indicating shorter times for the optimized configuration. To control the family-wise error rate, *p*-values were adjusted with the Holm procedure within each experiment family, at a significance level of α = 0.05. The *n* = 144 pairs per area pool all network sizes and repetitions. This is a deliberate blocked design: each pair is formed within one (network size, repetition) block on an identical node deployment, and the signed-rank test evaluates whether the optimization improves the metric consistently across the studied range of network sizes, without assuming a homogeneous effect magnitude. Because time effects nevertheless grow with network size, all headline comparisons were additionally verified on disjoint terciles of network size (Section 3.6). All analyses were performed with Python scripts (pandas v3.0.5, SciPy), which are available together with the raw simulation data (see the Data Availability Statement).

In addition to the primary campaign, four sets of supplementary experiments (464 runs in total) were performed with the same fixed parameters unless stated otherwise. First, for a quantitative validation against the original scheme, the baseline configuration was run at the operating point reported by Li et al. [33] (100 m × 100 m area, 140 nodes of which 40 anchors) and across anchor ratios of 10–70% (100 nodes), with five independent seeds each. Second, to test the sensitivity of the recommended neighborhood size to anchor density, the combined configuration (sweep line + local Voronoi, *k* ∈ {8, 10, 12, 16}) was compared with the original algorithm at anchor ratios of 20% and 60% (200 m × 200 m area, 100–900 nodes, three independent seeds). Third, to verify that the findings do not depend on the single generator stream of the primary campaign, the original and the combined configuration (*k* = 10 and 12) were re-run with ten independent seeds at 200, 600 and 1000 nodes. Fourth, to probe the sensitivity of the conclusions to the idealized strongest-anchor selection, a noise experiment perturbed the anchor selection with log-normal shadowing: the unknown node selects the anchor minimizing the path-loss equivalent 10*η*·log_10_(*d*) + *X_σ_*, with path-loss exponent *η* = 3 and *X_σ_* ~ *N*(0, *σ*^2^) dB for *σ* ∈ {4, 8}. The noise affects only the selection of the strongest-signal anchor (connectivity remains geometric), it is drawn from a dedicated generator so that node deployments and grouping remain identical, and the same noise realization is applied to all configurations of a given seed, preserving the paired design; it covers *N* ∈ {200, 600, 1000} in the 200 m area (three seeds) and the operating point of [33] (five seeds). All supplementary comparisons are paired by (seed, network size) and analyzed with the same statistical procedure as the primary campaign.

## 3. Results

The complete dataset comprises 5184 primary simulation runs (plus 464 supplementary runs described in Section 2.2): 48 network sizes (60–1000 nodes in steps of 20), three repetitions per configuration, two deployment areas (200 m × 200 m and 400 m × 400 m), all four combinations of the intersection algorithm (grid scanning vs. sweep line) and the Voronoi computation mode (global vs. local), and eight values of the neighborhood parameter *k* (6–20 in steps of 2) for the local mode. Unless stated otherwise, reported percentages are means over all network sizes of the per-size ratios between the optimized and the baseline configuration, and all statistical comparisons are paired two-sided Wilcoxon signed-rank tests over the 144 (network size, repetition) pairs with Holm correction within each experiment family.

### 3.1. Simulator Validation

Before evaluating the proposed optimizations, we verified that the simulator reproduces the qualitative behavior of the original VTL scheme reported by Li et al. [33]. Table 1 summarizes the baseline configuration (grid scanning, global Voronoi, 200 m × 200 m area). The normalized localization error *E_a_* decreases markedly with node density (Spearman ρ = −0.993, *p* < 0.001), from approximately 0.51 in the smallest networks to 0.12 at 1000 nodes, while the percentage of localized nodes *P_e_* increases with density (ρ = 0.883, *p* < 0.001) and saturates at 100% for networks of 500 nodes and above. Both trends match the behavior of the original algorithm, confirming that the simulator provides a valid baseline for the comparisons that follow.

A quantitative comparison at the operating point reported by Li et al. [33] (100 m × 100 m, 140 nodes of which 40 anchors, *R* = 20 m, *K* = 6) yields *E_a_* = 0.247 ± 0.012 and *P_e_* = 98.0 ± 2.3% over five independent seeds, against *E_a_* = 0.361 and *P_e_* = 100% reported for the original MATLAB implementation (MATLAB version not reported in [33]). Our simulator thus reproduces the qualitative behavior of the scheme with a somewhat lower absolute error. The discrepancy is expected: the simulator models anchor detection and nearest-anchor selection idealistically (no GPS positioning error and no propagation irregularities; see Section 4.6) and implements the grid test with a region–cell intersection predicate (Section 2.1). Since all four evaluated configurations share the identical implementation of every phase, only their relative behavior is interpreted in this paper, and a conservative baseline does not favor the optimized variants. The noise experiment introduced in Section 2.2 supports this interpretation quantitatively: with log-normal shadowing applied to the anchor selection, the error at the operating point of [33] increases from *E_a_* = 0.247 ± 0.012 (no noise) to 0.286 ± 0.031 at *σ* = 4 dB and 0.322 ± 0.023 at *σ* = 8 dB, approaching the value of 0.361 reported in [33], while *P_e_* remains 98.0 ± 2.3%. The lower baseline error of our simulator is therefore attributable to its noise-free RSSI model rather than to a difference in the algorithm itself. The dependence on anchor density also matches the original report: across anchor ratios of 10–70% (100 nodes, five seeds), *E_a_* decreased monotonically from 0.54 ± 0.02 to 0.16 ± 0.02 while *P_e_* rose from 62% to 100% (Spearman ρ = −0.98 and 0.92, respectively; both *p* < 0.001), mirroring the trends reported in Figure 11 of [33].

### 3.2. Experiment 1: Local vs. Global Voronoi Computation

**Figure 4:**
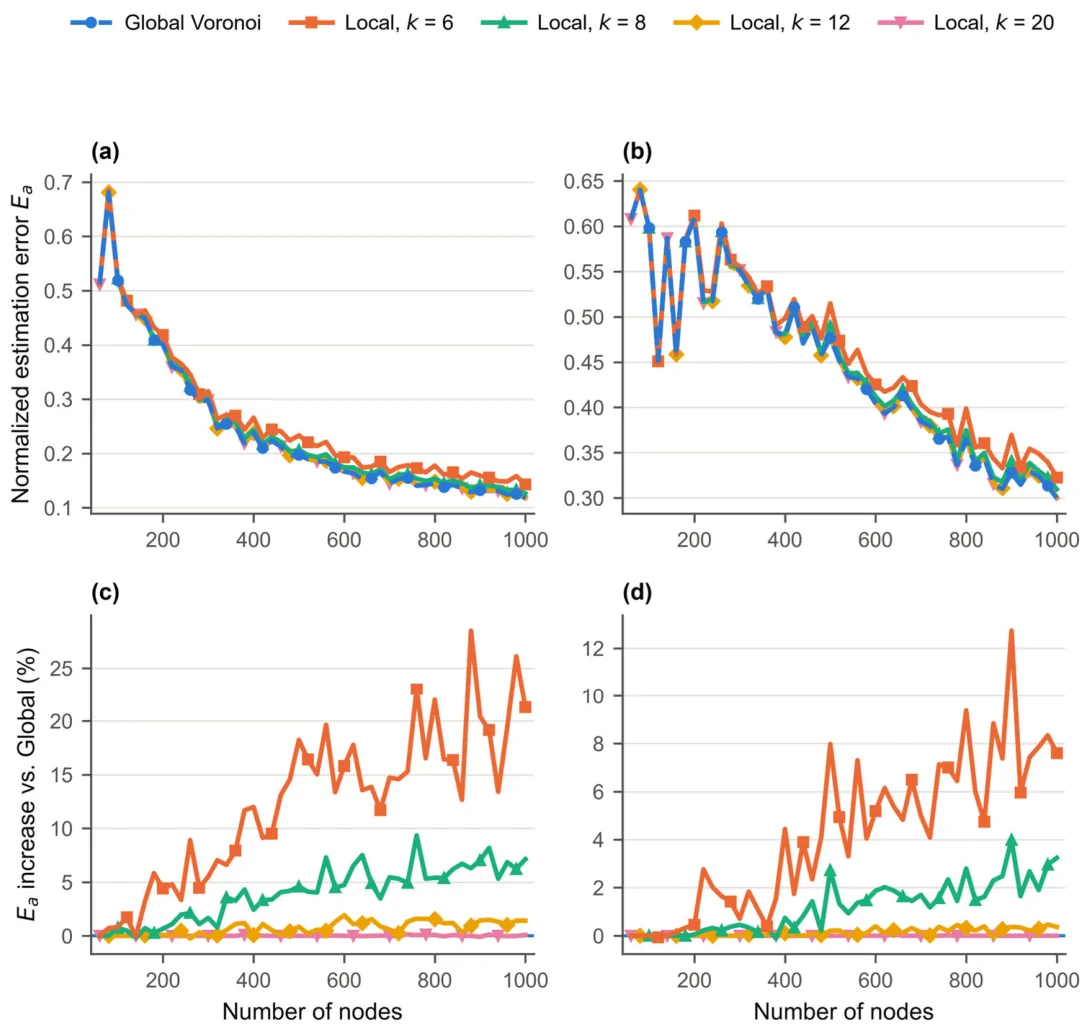
Accuracy of the local Voronoi computation (grid scanning configurations): (**a**,**b**) normalized estimation error *E_a_* as a function of network size for the global baseline and the local variant with *k* = 6, 8, 12 and 20, in the (**a**) 200 m × 200 m and (**b**) 400 m × 400 m areas; (**c**,**d**) the same data shown as the relative increase in *E_a_* with respect to the global baseline (dashed line at zero), in the (**c**) 200 m and (**d**) 400 m areas. For legibility, 4 of the 8 tested values of *k* are plotted; the remaining curves fall between the ones shown (see Table 2).

**Figure 5:**
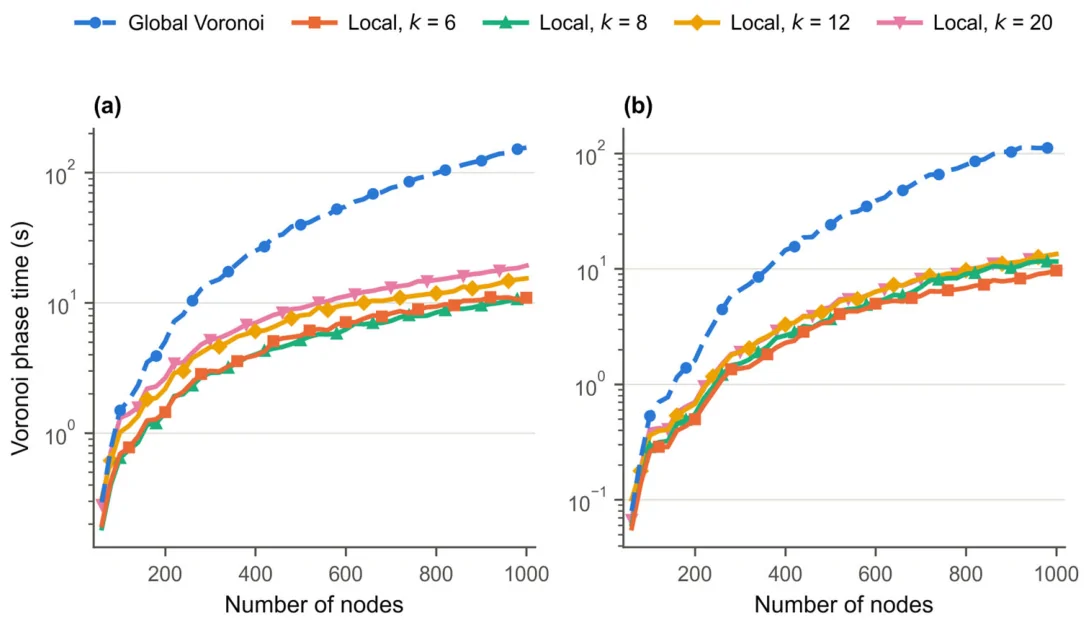
Execution time of the Voronoi construction phase as a function of network size (logarithmic time scale): (**a**) 200 m × 200 m area; (**b**) 400 m × 400 m area. For legibility, 4 of the 8 tested values of *k* are plotted; the remaining curves fall between the ones shown (see Table 2).

Figure 4a,b shows *E_a_* as a function of network size for the global Voronoi baseline and the local variant with *k* = 6, 8, 12 and 20, and Figure 4c,d shows the same data as the relative increase in *E_a_* with respect to the global baseline; Figure 5 shows the corresponding Voronoi-phase execution times. Table 2 quantifies the effect for every value of *k*.

The local approach reduced the Voronoi-phase time by 69–83% depending on *k* and the deployment area, with large and consistent effect sizes (*d_z_* ≈ −1.0 for all comparisons). In absolute terms, at 1000 nodes in the 200 m area the Voronoi phase took 155.7 s with the global approach versus 10.5 s with the local approach at *k* = 8, a roughly fifteen-fold reduction.

**Figure 6:**
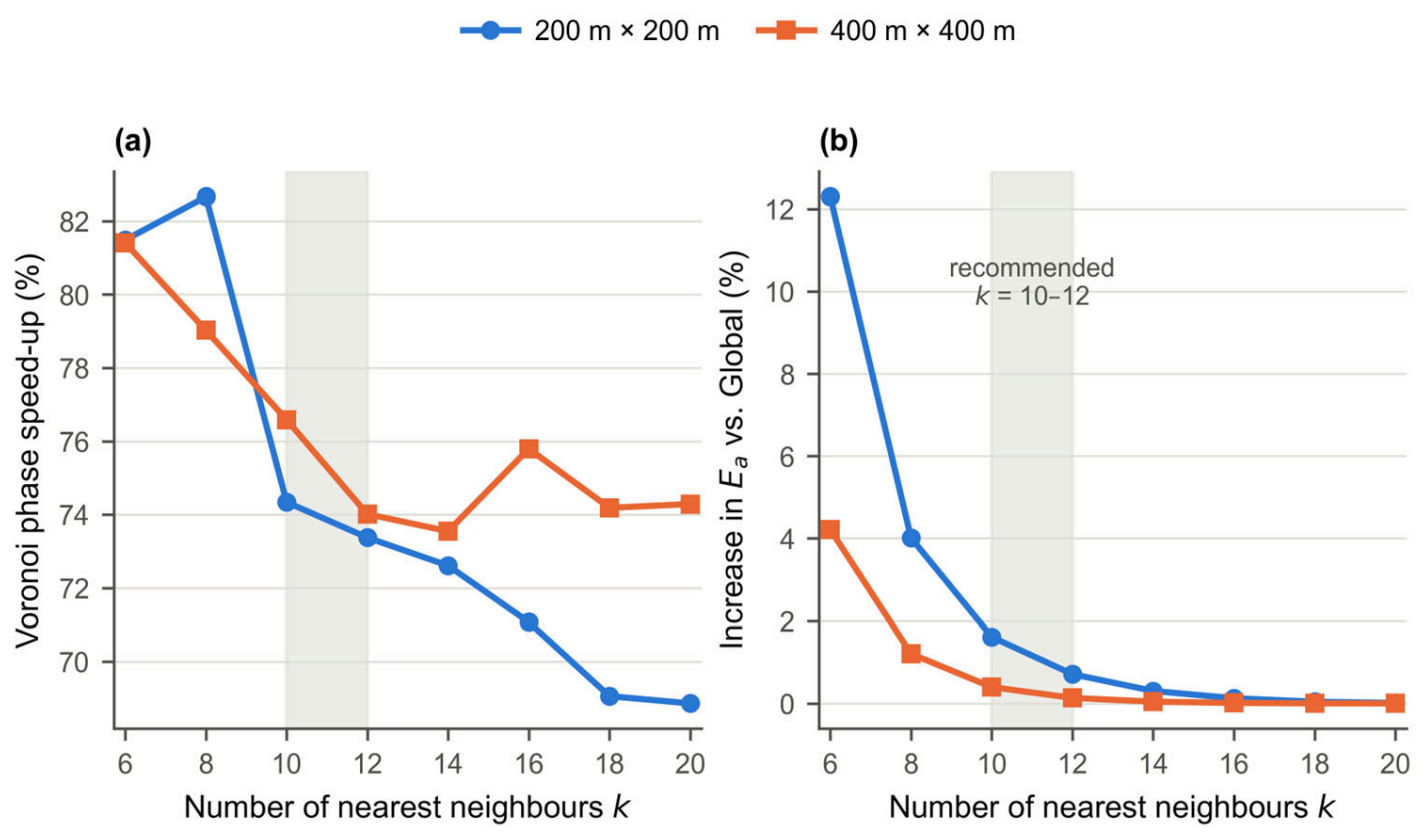
Accuracy–speed trade-off of the local Voronoi computation across all tested values of *k*: (**a**) speed-up of the Voronoi phase; (**b**) relative increase in *E_a_* with respect to the global baseline. The shaded band marks the recommended operating range *k* = 10–12.

The impact on accuracy, however, depends strongly on *k*. For *k* = 6 the error increase is substantial (+12.3% in the 200 m area) and statistically significant with a very large effect size (*d_z_* = 2.51); it drops to +4.0% for *k* = 8, +1.6% for *k* = 10, and below +1% for *k* ≥ 12, becoming practically negligible (≤0.1%) for *k* ≥ 16. In the sparser 400 m area the same pattern appears with smaller magnitudes (+4.2% at *k* = 6, ≤0.4% for *k* ≥ 10). Although even the smallest differences remain statistically detectable (a consequence of the paired design, in which both configurations share identical node deployments), their practical relevance vanishes for *k* ≥ 10. Figure 6 summarizes this accuracy–speed trade-off across all tested *k*.

The percentage of localized nodes *P_e_* was completely unaffected by the choice of Voronoi mode: the paired differences were exactly zero for every *k*, every network size and both areas. The local approximation therefore influences only *where* a node is estimated to be, not *whether* it can be localized.

### 3.3. Experiment 2: Sweep Line vs. Grid Scanning

**Figure 7:**
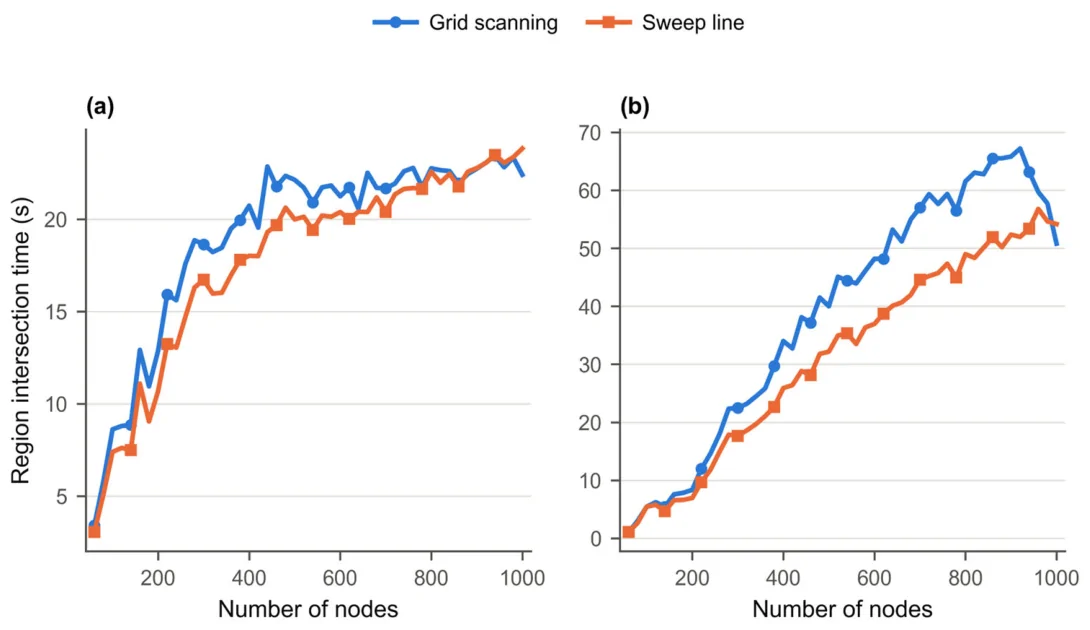
Execution time of the positive-region intersection phase as a function of network size, for grid scanning and the sweep line algorithm (global Voronoi configurations): (**a**) 200 m × 200 m area; (**b**) 400 m × 400 m area.

Figure 7 and Table 3 compare the two algorithms for the positive-region intersection phase on the global-Voronoi configurations.

The sweep line algorithm reduced the intersection-phase time by 7.7% in the 200 m area and by 18.1% in the 400 m area, translating into total-time reductions of 3.8% and 10.2%, respectively. The advantage grows with the deployment area, as expected: the cost of grid scanning is proportional to the number of grid cells, which grows quadratically with the linear dimension of the area at a fixed resolution, whereas the sweep line touches only the grid columns and rows actually spanned by the positive regions.

Crucially, the sweep line algorithm is an exact replacement: the localization results were identical to those of grid scanning. Across all 288 paired runs in both areas, the differences in *E_a_* and in *P_e_* were exactly zero for every pair. The optimization therefore affects execution time only, with no approximation involved.

### 3.4. Experiment 3: Combined Optimizations

**Figure 8:**
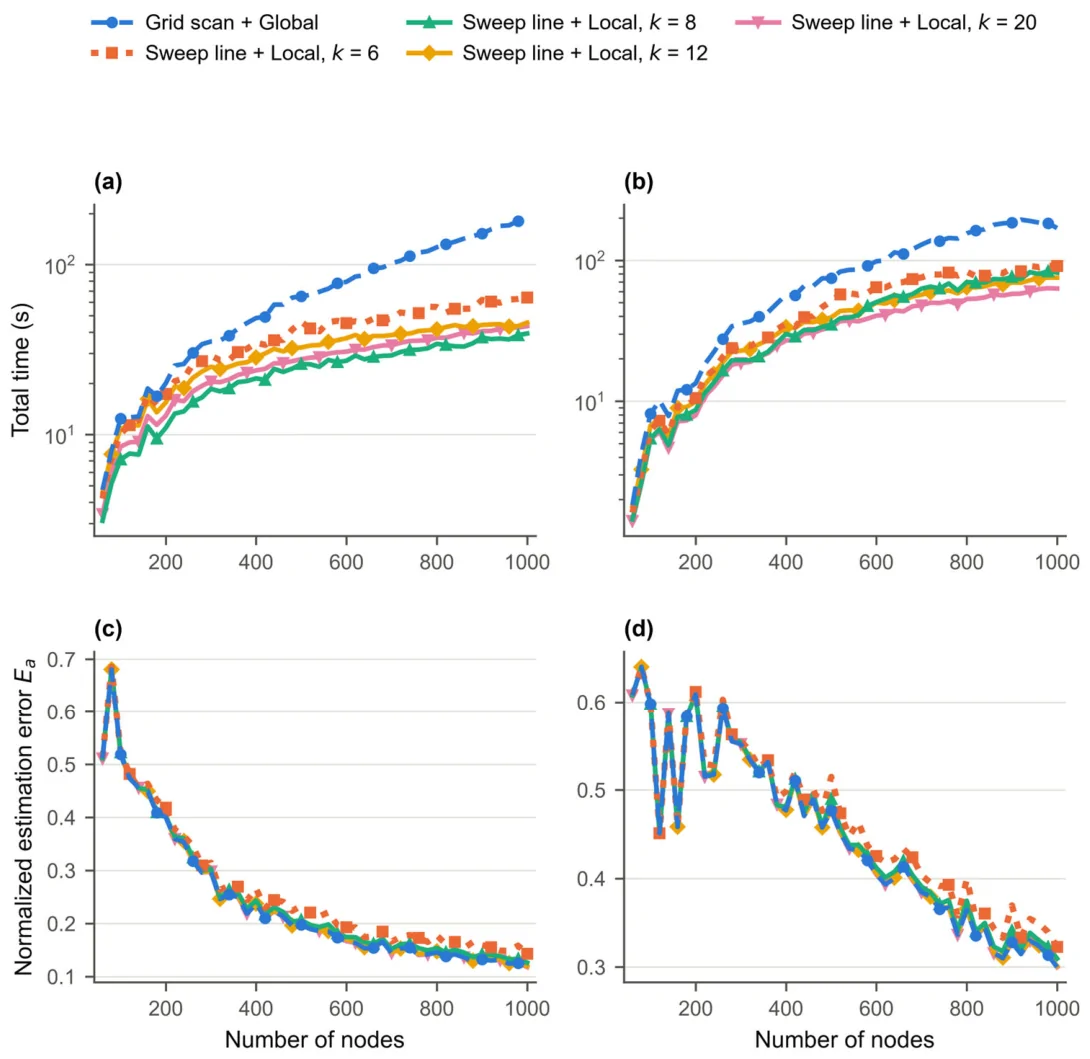
Combined optimizations (sweep line + local Voronoi with *k* = 6, 8, 12 and 20) vs. the original algorithm (grid scanning + global Voronoi): (**a**,**b**) total execution time (logarithmic scale); (**c**,**d**) average normalized error *E_a_*; left column (**a**,**c**) 200 m × 200 m area, right column (**b**,**d**) 400 m × 400 m area. The pathological case *k* = 6 is drawn with a thicker dotted line to distinguish it from the recommended settings (solid lines) and the baseline (dashed line).

The two optimizations address different phases of the algorithm and can be applied together. Figure 8 and Table 4 compare the fully optimized configuration (sweep line + local Voronoi) against the original algorithm (grid scanning + global Voronoi).

The combined configuration reduced the total execution time by 37–61% depending on *k* and the area. At 1000 nodes in the 200 m area, the total time dropped from 183.9 s for the original algorithm to 39.3 s for the combined configuration with *k* = 8 (45.5 s with *k* = 12), a 4–4.7-fold reduction.

An unexpected result concerns *k* = 6: although its Voronoi phase is among the fastest (Table 2), its total-time speed-up (37.9% at 200 m) is markedly lower than for any larger *k*. The cause lies in the intersection phase: with too few neighbors the local Voronoi diagram approximates the true nearest-node cell poorly, producing larger positive regions that the intersection phase must subsequently process. In the 200 m area the mean intersection-phase time was 29.4 s for *k* = 6, versus 16.1 s for *k* = 8 and 18.9 s for *k* = 12 (36.9, 29.3 and 27.4 s, respectively, in the 400 m area). The choice *k* = 6 therefore degrades both accuracy and overall performance, and is dominated by every larger tested value of *k* on both criteria.

### 3.5. Choice of k

Combining the results of Experiments 1 and 3 (Figure 6), the parameter *k* exhibits a clear sweet spot. Values *k* = 10–12 retain 73–77% of the Voronoi-phase speed-up while keeping the accuracy penalty below 2% (below 0.5% in the sparser area), and deliver 45–56% total-time reductions in the combined configuration. The value *k* = 8 maximizes the combined speed-up (60.8% at 200 m) at the cost of a +4% error increase, which may be acceptable when execution time is the primary constraint. The value *k* = 6 should be avoided altogether, and values *k* ≥ 14 bring no further accuracy benefit while slightly increasing the Voronoi-phase cost. We therefore recommend *k* = 10–12 as the default operating range.

### 3.6. Robustness to Anchor Density, Seed Variability and RSSI Noise

The recommendation *k* = 10–12 was derived at the default anchor ratio of 40%. Table 5 examines whether it survives at a substantially lower (20%) and higher (60%) anchor density, comparing the combined configuration against the original algorithm over 100–900 nodes with three independent seeds (*n* = 27 pairs per comparison). At an anchor ratio of 20%, the accuracy penalty of the local computation shrinks (at most +0.8% for *k* ≥ 10) and the combined optimizations reduce the total execution time by 24–30%. At 60%, the penalty grows for small neighborhoods (+6.4% at *k* = 8 and +3.0% at *k* = 10), whereas *k* = 12 remains at +1.3% and *k* = 16 at +0.3%, with total-time reductions of 54–57%. The percentage of localized nodes was again identical in every pair. The accuracy cost of a given *k* therefore increases with anchor density (in denser anchor sets, the nearest cell has more Delaunay neighbors, so a fixed-size neighborhood more often omits one), and the upper end of the recommended range should be preferred in anchor-dense networks. All differences in Table 5 are statistically significant (paired two-sided Wilcoxon tests, Holm-corrected within the experiment family).

Table 6 addresses the dependence on the pseudo-random seed. The original and the combined configuration (*k* = 10 and 12) were re-run with ten independent seeds at three network sizes. The relative total-time reductions are tightly concentrated (72.9 ± 2.3% at 1000 nodes for *k* = 12), the percentage of localized nodes was identical in all 60 pairs, and the accuracy penalty of *k* = 12 stayed within 2% in every setting. The penalty of *k* = 10 at the largest, densest network (+3.0% at 1000 nodes) exceeds the campaign-average figure, consistent with the density effect observed in Table 5; the primary conclusions are therefore not an artefact of the single generator stream used in the main campaign.

Under shadowing noise the baseline error increases substantially (for example, at *N* = 1000 from *E_a_* = 0.122 without noise to 0.189 at *σ* = 4 dB and 0.257 at *σ* = 8 dB), while the percentage of localized nodes is unaffected, confirming that the idealized anchor selection is the dominant idealization of the simulator. The accuracy penalty of the local approximation, however, does not compound with the noise: the paired *E_a_* difference between the fully optimized configuration and the baseline slightly decreases with noise, from +0.86% (*σ* = 0) to +0.60% (*σ* = 4 dB) and +0.49% (*σ* = 8 dB) for *k* = 12, and from +1.74% to +1.06% and +0.84% for *k* = 10, and *P_e_* remains identical in every pair. The interaction between RSSI noise and the local approximation thus acts in the benign direction: once the anchor selection is perturbed by noise, the small geometric perturbation introduced by the local diagram becomes relatively less important.

Finally, to verify that the pooled tests are not driven by any particular range of network sizes, the four headline comparisons (Voronoi-phase time and *E_a_* for local *k* = 12 vs. global; intersection-phase time for sweep line vs. grid scanning; total time for the combined configuration) were repeated on disjoint terciles of network size (60–360, 380–680 and 700–1000 nodes; 48 pairs each, Holm-corrected within each family). The direction of every effect is preserved in every tercile and both areas, and 23 of the 24 tests remain significant. The magnitudes follow the expected scaling: the Voronoi-phase reduction grows from 47–50% in the smallest tercile to about 88% in the largest, the total-time reduction grows from 14–21% to 58–67%, and the accuracy penalty of *k* = 12 remains below 1.2% in all terciles. The single exception is the intersection-phase comparison in the 200 m area for the largest networks (−0.6%, not significant), consistent with the convergence of the two curves in Figure 7a; in the 400 m area the sweep-line advantage persists across all terciles (14–22%).

## 4. Discussion

The aim of this work was to improve the computational efficiency of the VTL localization algorithm through two optimizations (a local Voronoi computation and a sweep line replacement for grid scanning) without compromising its localization quality. The discussion is organized around the four hypotheses stated in the Introduction.

### 4.1. H1: The Local Voronoi Approach Significantly Reduces Execution Time

Hypothesis H1 is confirmed, and the effect is considerably larger than suggested by earlier preliminary experiments. The local computation reduced the Voronoi-phase time by 69–83% across all tested values of *k* and both deployment areas, with uniformly large effect sizes; at 1000 nodes the phase was up to fifteen times faster in absolute terms. The result empirically confirms the geometric intuition behind the optimization: the shape of the Voronoi cell relevant for localization is determined solely by the immediate neighbors of the nearest anchor, so excluding distant anchors removes computational work without changing the geometry that matters; provided enough neighbors are included, which leads directly to H2.

### 4.2. H2: The Parameter k Trades Accuracy for Speed, but Not Monotonically

Hypothesis H2 is confirmed in its accuracy component and refined in its time component. As expected, increasing *k* monotonically reduces the localization error toward the global baseline: the error surplus falls from +12.3% at *k* = 6 to below +1% at *k* ≥ 12 and effectively zero at *k* ≥ 16 (200 m area), with the same pattern at smaller magnitudes in the sparser 400 m area.

Two findings, however, qualify the simple “larger *k* costs more time” picture. First, the accuracy penalty at *k* = 6 is far from negligible, contradicting the assumption (common when the neighborhood is chosen ad hoc) that any local approximation preserves accuracy. Second, and more surprisingly, a too-small *k* also hurts overall performance: the poorly approximated (larger) positive regions produced at *k* = 6 nearly double the intersection-phase workload compared with *k* = 8. The total execution time is therefore non-monotonic in *k*, and *k* = 6 is dominated on both criteria by every larger tested value. The cost of the local approximation thus propagates downstream through the algorithm pipeline, an interaction that could not have been observed by benchmarking the Voronoi phase in isolation.

Based on the joint accuracy–speed trade-off we recommend *k* = 10–12 as the default operating range (error within 2% of the global baseline, 73–77% Voronoi-phase and 45–56% total-time reduction), with *k* = 8 as an option when execution time is the primary constraint and a ~4% error increase is acceptable. Since network density affects the magnitude of the accuracy penalty, an adaptive selection of *k* based on local anchor density is a natural direction for future work.

### 4.3. H3: The Sweep Line Algorithm Accelerates the Intersection Phase Without Any Loss of Accuracy

Hypothesis H3 is confirmed in a stronger form than originally stated. The sweep line algorithm reduced the intersection-phase time by 7.7% (200 m) and 18.1% (400 m), and the total time by 3.8% and 10.2%, respectively. More importantly, the replacement is exact: across all 288 paired runs both the normalized localization error and the percentage of localized nodes were identical to the grid scanning results, pair by pair. The optimization is therefore a pure implementation improvement with no algorithmic trade-off.

The dependence on the deployment area follows from the asymptotic behavior of the two approaches. At a fixed grid resolution, the number of cells that grid scanning must test grows quadratically with the linear dimension of the area (i.e., linearly with its area), regardless of the network topology, whereas the sweep-line workload scales with the number of grid cells actually spanned by the positive regions. The advantage of the sweep line approach is consequently largest exactly where the original algorithm is weakest (large, sparse deployment areas), which is also the regime most relevant for real-world WSN deployments. The tercile analysis in Section 3.6 adds one qualification: in the smaller 200 m area the sweep-line advantage diminishes with network density and is no longer significant in the largest tercile, so the benefit of the sweep line is most pronounced in larger or sparser deployments.

### 4.4. H4: The Combined Optimizations Preserve Accuracy While Substantially Improving Scalability

Hypothesis H4 is confirmed. Applied together, the two optimizations reduced the total execution time by 37–61% (up to 4.7-fold at 1000 nodes), while the accuracy cost remains fully determined by the choice of *k*, since the sweep line component introduces no error. With the recommended *k* = 10–12, the combined algorithm runs roughly twice as fast overall with an error within 2% of the original, and the percentage of localized nodes is unchanged in all configurations. Because the two optimizations target different phases (diagram construction and region intersection), their benefits compose without interference, except for the beneficial interaction already noted: a sufficiently large *k* also keeps the intersection phase efficient.

### 4.5. Practical Implications

For practitioners, the results translate into simple guidance, with the caveat that it is derived from uniform deployments in square areas and should be read as a default operating range rather than a universal constant. The local Voronoi optimization should be enabled with *k* in the 10–12 range, preferring the upper end in densely anchored networks: the accuracy penalty of a fixed *k* grows with anchor density and network size (up to about +3% for *k* = 10 in the densest settings, Section 3.6), while the geometric analysis in Section 2.1 explains why the range itself is stable: the expected number of Delaunay neighbours (about six) does not grow with density. Where density is unknown or variable, a simple density-aware rule (*k* near 10 in sparse regions, 12–16 in dense ones) follows directly from Table 2 and Table 5, and a fully adaptive selection remains future work (Section 4.7); the sweep line algorithm should always replace grid scanning, as it is exact and was never slower in our experiments; and *k* = 6, although geometrically sufficient to define a cell in the average case, should be avoided as it degrades both accuracy and speed. The reduced computational load directly benefits the energy budget of the nodes performing localization, which is often the binding constraint in WSN deployments.

This study deliberately refrains from re-comparing the optimized algorithm with other localization schemes. The sweep line replacement is exact, and the local Voronoi computation changes the estimates by less than 2% at the recommended settings, so the localization accuracy of the optimized VTL is, by construction, that of the original scheme, which was already compared favorably with Centroid and APIT in [33]. The contribution of this paper is orthogonal to such comparisons: it reduces the computational cost of an existing scheme without altering its localization behavior.

Regarding deployment on resource-constrained hardware, three observations support the transfer of the reported gains. First, both optimizations reduce the number of geometric operations rather than exploit any property of the desktop platform: the local computation constructs diagrams over *k* instead of *m* sites and post-processes *Θ*(*k*) instead of *Θ*(*m*) cell polygons, and the sweep is output-sensitive in the number of grid cells actually covered (Section 2.1); profiling confirms that the measured speed-ups correspond to proportional reductions in the number of calls to identical geometric primitives (Section 2.2). Such operation-count reductions carry over to any implementation, including compiled code on embedded processors, even though absolute times will differ. Second, the optimizations also reduce the memory footprint (the local diagram stores *k* instead of up to *m* sites and their cells), which on memory-limited nodes is at least as important as time. Third, they require no additional communication or information beyond what the VTL protocol already provides (the anchor list and received signal strengths), so they are drop-in replacements at the algorithmic level. An implementation study on embedded hardware, with measured energy consumption, remains future work (Section 4.7).

### 4.6. Limitations

Several limitations qualify the scope of these findings. First, nodes were deployed uniformly at random in a square area; clustered or obstacle-constrained topologies may change the optimal *k*. Second, the primary campaign models the RSSI-based nearest-anchor selection idealistically, without a radio propagation or noise model, so the reported errors isolate the geometric behavior of the algorithm rather than end-to-end accuracy in a physical deployment. The supplementary shadowing experiment (Section 2.2 and Section 3.6) mitigates this concern: noise mainly shifts the absolute error level (and explains the gap to the operating point of [33], Section 3.1), whereas the comparative conclusions about the optimizations are unchanged, with the local-approximation penalty even slightly decreasing under noise. A full propagation model (fading, obstacles, EGPS-like corrections) remains future work. Third, execution times were measured on a single machine (Section 2.2) in a single-threaded Python implementation; absolute times would differ on embedded hardware, although the relative comparisons (which rely on identical implementations of the shared phases) should transfer. Fourth, while the anchor ratio was varied in the validation (10–70%) and robustness (20% and 60%) experiments, the number of groups (*K* = 6) and the grid resolution (2 m) were held fixed; their interaction with the proposed optimizations remains to be explored. Finally, the primary campaign used three repetitions drawn from one generator stream; the ten-seed verification in Section 3.6 confirms the stability of the reported effects, but the absolute error levels would still vary with other deployments.

### 4.7. Future Work

Beyond an adaptive, density-aware choice of *k* (a direction directly motivated by the sensitivity analysis in Section 3.6, which shows that the accuracy cost of a fixed *k* grows with anchor density), future work includes validating the optimized algorithm on physical WSN testbeds with real radio propagation (including interference from heterogeneous technologies sharing the same unlicensed spectrum, whose coexistence dynamics are an active research area in adjacent wireless domains [42]), extending the analysis to non-uniform deployments, quantifying the energy savings on embedded hardware, and parallelizing the per-group computations, which are naturally independent.

## 5. Conclusions

This paper addressed the computational efficiency of the VTL localization algorithm in wireless sensor networks. An analysis of the original scheme identified two computationally expensive steps: the repeated global construction of Voronoi diagrams for every group of anchor nodes, and the brute-force grid scan used to determine the intersection of the positive regions. Two optimizations were proposed and implemented: a local Voronoi computation restricted to the *k* anchors nearest to the strongest-signal anchor, and an exact sweep line algorithm for the intersection phase.

Both optimizations were evaluated in a purpose-built, reproducible Python simulator through 5184 primary and 464 supplementary simulations covering networks of 60–1000 nodes, two deployment areas and eight values of *k*, with paired statistical tests. The local Voronoi computation reduced the Voronoi-phase time by 69–83%, with an accuracy penalty below 2% for *k* ≥ 10 on average and no effect whatsoever on the percentage of localized nodes. The sweep line algorithm reduced the intersection-phase time by 7.7–18.1% (3.8–10.2% of the total time) while producing localization results identical to grid scanning. Combined, the two optimizations reduced the total execution time by 37–61% (up to 4.7-fold at 1000 nodes), making the VTL algorithm considerably more practical for large networks and resource-constrained hardware.

Beyond the raw speed-ups, the analysis yielded two findings of practical value. The neighborhood size *k* = 10–12 is recommended as the default operating range, balancing accuracy and efficiency. Conversely, an overly aggressive reduction of the neighborhood (*k* = 6) is counterproductive: it degrades both accuracy and total execution time, because the poorly approximated positive regions it produces increase the workload of the subsequent intersection phase.

In addition to the optimizations themselves, a contribution of this work is the openly available simulator and dataset, which allow all reported results to be reproduced and provide a platform for future research, including adaptive neighborhood selection and validation on physical WSN testbeds.

## Figures and Tables

**Table 1:** Baseline behavior of the simulated VTL algorithm (grid scanning + global Voronoi, 200 m × 200 m area; means over three repetitions).

Number of Nodes	60	100	200	400	600	800	1000
*E* *_a_*	0.511	0.519	0.401	0.237	0.167	0.146	0.118
*P_e_* (%)	41.7	80.0	81.7	96.7	100.0	100.0	100.0

**Table 2:** Local vs. global Voronoi computation (grid scanning configurations): speed-up of the Voronoi phase and relative increase in *E_a_*; paired Wilcoxon tests (*n* = 144, Holm-corrected).

Area	*k*	Voronoi Speed-Up (%)	Δ*E_a_* (%)	*p* (Time)	*d_z_* ^1^ (Time)	*p* (*E_a_*)	*d_z_* ^1^ (*E_a_*)
200 m	6	81.5	+12.30	<0.001	−1.07	<0.001	2.51
200 m	8	82.7	+4.01	<0.001	−1.08	<0.001	2.15
200 m	10	74.3	+1.61	<0.001	−1.07	<0.001	1.49
200 m	12	73.4	+0.71	<0.001	−1.05	<0.001	1.21
200 m	14	72.6	+0.30	<0.001	−1.05	<0.001	0.90
200 m	16	71.1	+0.13	<0.001	−1.04	<0.001	0.79
200 m	18	69.1	+0.04	<0.001	−1.04	<0.001	0.55
200 m	20	68.9	+0.01	<0.001	−1.03	<0.001	0.33
400 m	6	81.4	+4.21	<0.001	−0.99	<0.001	1.52
400 m	8	79.0	+1.21	<0.001	−1.00	<0.001	1.27
400 m	10	76.6	+0.40	<0.001	−0.99	<0.001	1.15
400 m	12	74.0	+0.13	<0.001	−0.98	<0.001	0.94
400 m	14	73.5	+0.04	<0.001	−0.97	<0.001	0.65
400 m	16	75.8	+0.01	<0.001	−0.98	<0.001	0.48
400 m	18	74.2	+0.00	<0.001	−0.98	<0.001	0.32
400 m	20	74.3	+0.00	<0.001	−0.98	0.002	0.22

^1^ For paired designs, |*d_z_*| values of about 0.2, 0.5 and 0.8 are conventionally interpreted as small, medium and large effects, respectively.

**Table 3:** Sweep line vs. grid scanning (global Voronoi configurations); paired Wilcoxon tests (*n* = 144, Holm-corrected).

Area	Metric	Speed-Up (%)	*p*	*d_z_* ^1^
200 m	Intersection phase	7.7	<0.001	−0.98
200 m	Total execution time	3.8	<0.001	−0.39
400 m	Intersection phase	18.1	<0.001	−1.20
400 m	Total execution time	10.2	<0.001	−0.67

^1^ For paired designs, |*d_z_*| values of about 0.2, 0.5 and 0.8 are conventionally interpreted as small, medium and large effects, respectively.

**Table 4:** Combined optimizations vs. the original algorithm; paired Wilcoxon tests (*n* = 144, Holm-corrected). Δ*E_a_* values are identical to Table 2 because the sweep line component does not affect accuracy.

Area	*k*	Total-Time Speed-Up (%)	Δ*E_a_* (%)	*p* (Time)	*d_z_* ^1^ (Time)
200 m	6	37.9	+12.30	<0.001	−0.90
200 m	8	60.8	+4.01	<0.001	−1.15
200 m	10	55.8	+1.61	<0.001	−1.13
200 m	12	47.0	+0.71	<0.001	−0.98
200 m	14	55.5	+0.30	<0.001	−1.11
200 m	16	56.9	+0.13	<0.001	−1.12
200 m	18	56.2	+0.04	<0.001	−1.12
200 m	20	55.4	+0.01	<0.001	−1.11
400 m	6	37.1	+4.21	<0.001	−0.85
400 m	8	48.5	+1.21	<0.001	−1.17
400 m	10	36.6	+0.40	<0.001	−0.89
400 m	12	44.9	+0.13	<0.001	−1.06
400 m	14	45.2	+0.04	<0.001	−1.06
400 m	16	51.2	+0.01	<0.001	−1.07
400 m	18	55.1	+0.00	<0.001	−1.14
400 m	20	54.7	+0.00	<0.001	−1.13

^1^ For paired designs, |*d_z_*| values of about 0.2, 0.5 and 0.8 are conventionally interpreted as small, medium and large effects, respectively.

**Table 5:** Sensitivity of the combined optimizations to the anchor ratio (200 m × 200 m area, 100–900 nodes, three seeds; sweep line + local Voronoi vs. the original algorithm; paired Wilcoxon tests, *n* = 27, Holm-corrected).

Anchor Ratio	*k*	Δ*E_a_* (%)	Voronoi Speed-Up (%)	Total-Time Speed-Up (%)	*p* (*E_a_*)	*d_z_* ^1^ (*E_a_*)	*p* (Time)	*d_z_* ^1^ (Time)
20%	8	+2.40	57.6	27.6	<0.001	1.56	<0.001	−1.24
20%	10	+0.80	53.3	29.7	<0.001	1.34	<0.001	−1.29
20%	12	+0.29	49.3	27.6	<0.001	1.30	<0.001	−1.11
20%	16	+0.04	41.2	23.9	0.002	0.65	<0.001	−0.98
60%	8	+6.38	80.2	54.4	<0.001	2.15	<0.001	−0.92
60%	10	+2.95	78.7	57.3	<0.001	1.67	<0.001	−0.93
60%	12	+1.28	76.4	56.5	<0.001	1.27	<0.001	−0.91
60%	16	+0.25	72.3	54.4	<0.001	0.91	<0.001	−0.90

^1^ For paired designs, |*d_z_*| values of about 0.2, 0.5 and 0.8 are conventionally interpreted as small, medium and large effects, respectively.

**Table 6:** Stability of the combined optimizations over ten independent seeds (200 m × 200 m area, 40% anchors; mean ± SD over seeds; paired by seed).

*k*	Nodes	*E_a_* Original	*E_a_* Combined	Δ*E_a_* (%)	Total-Time Speed-Up (%)
10	200	0.348 ± 0.030	0.350 ± 0.030	+0.41	21.5 ± 11.5
10	600	0.172 ± 0.008	0.175 ± 0.008	+1.69	56.9 ± 3.4
10	1000	0.124 ± 0.004	0.128 ± 0.004	+3.03	72.5 ± 1.2
12	200	0.348 ± 0.030	0.349 ± 0.030	+0.12	23.3 ± 5.8
12	600	0.172 ± 0.008	0.173 ± 0.008	+0.89	56.9 ± 3.2
12	1000	0.124 ± 0.004	0.126 ± 0.004	+1.47	72.9 ± 2.3

## Data Availability

The source code of the simulator, the analysis scripts and the complete simulation dataset (5184 primary and 464 supplementary runs, CSV) are openly available on GitHub at https://github.com/gdambic/vtl-localization-optimizations (accessed on 1 October 2026) and are permanently archived on Zenodo at https://doi.org/10.5281/zenodo.23076732.

## Abbreviations

The following abbreviations are used in this manuscript:

WSN	Wireless sensor network
GPS	Global positioning system
VTL	Voronoi diagram-based grouping test localization
RSSI	Received signal strength indication
AoA	Angle of arrival
TDoA	Time difference of arrival
ToA	Time of arrival
APIT	Approximate point in triangulation
